# Impact of brief prewarming on anesthesia-related core-temperature drop, hemodynamics, microperfusion and postoperative ventilation in cytoreductive surgery of ovarian cancer: a randomized trial

**DOI:** 10.1186/s12871-019-0828-1

**Published:** 2019-08-22

**Authors:** L. Kaufner, P. Niggemann, T. Baum, S. Casu, J. Sehouli, A. Bietenbeck, M. Boschmann, C. D. Spies, A. Henkelmann, C. von Heymann

**Affiliations:** 1 Department of Anaesthesiology and Operative Intensive Care Medicine (CCM, CVK), Charité-Universitätsmedizin Berlin, Corporate Member of Freie Universität Berlin, Humboldt-Universität zu Berlin, and Berlin Institute of Health, Campus Virchow-Klinikum, Augustenburger Platz 1, 13353 Berlin, Germany; 2 Department of Anaesthesia, Intensive Care Medicine, Emergency Medicine and Pain Therapy, Vivantes Klinikum im Friedrichshain, Berlin, Germany; 3 Department of Gynaecology, Charité-Universitätsmedizin Berlin, Berlin, Germany; 4 Institut für Klinische Chemie und Pathobiochemie, Klinikum rechts der Isar der Technischen Universität München, Munich, Germany; 5 Experimental & Clinical Research Center, ECRC, Charité-Universitätsmedizin Berlin CCB, Berlin, Germany

**Keywords:** Prewarming, Ovarian cancer, Microperfusion, Normothermia

## Abstract

**Background:**

General (GA)- and epidural-anesthesia may cause a drop in body-core-temperature (BCT_drop_), and hypothermia, which may alter tissue oxygenation (StO_2_) and microperfusion after cytoreductive surgery for ovarian cancer. Cell metabolism of subcutaneous fat- or skeletal muscle cells, measured in microdialysis, may be affected. We hypothesized that forced-air prewarming during epidural catheter placement and induction of GA maintains normothermia and improves microperfusion.

**Methods:**

After ethics approval 47 women scheduled for cytoreductive surgery were prospectively enrolled. Women in the study group were treated with a prewarming of 43 °C during epidural catheter placement. BCT (Spot on®, 3 M) was measured before (T_1_), after induction of GA (T_2_) at 15 min (T_3_) after start of surgery, and until 2 h after ICU admission (T_ICU2h_). Primary endpoint was BCT_drop_ between T_1_ and T_2_. Microperfusion-, hemodynamic- and clinical outcomes were defined as secondary outcomes. Statistical analysis used the Mann-Whitney-U- and non-parametric-longitudinal tests.

**Results:**

BCT_drop_ was 0.35 °C with prewarming and 0.9 °C without prewarming (*p* < 0.005) and BCT remained higher over the observation period (ΔT_4_ = 0.9 °C up to ΔT_7_ = 0.95 °C, *p* < 0.001). No significant differences in hemodynamic parameters, transfusion, arterial lactate and dCO_2_ were measured. In microdialysis the ethanol ratio was temporarily, but not significantly, reduced after prewarming. Lactate, glucose and glycerol after PW tended to be more constant over the entire period. Postoperatively, six women without prewarming, but none after prewarming were mechanical ventilated (*p* < 0.001).

**Conclusion:**

Prewarming at 43 °C reduces the BCT_drop_ and maintains normothermia without impeding the perioperative routine patient flow. Microdialysis indicate better preserved parameters of microperfusion.

**Trial registration:**

ClinicalTrials.gov; ID: NCT02364219; Date of registration: 18-febr-2015.

## Background

The incidence of inadvertent perioperative hypothermia in surgery, defined as a body core temperature (BCT) below 36 °C, is still high [1, 2]. A shift of thresholds for vasoconstriction and shivering as well as sympathicolysis-related vasodilatation caused by epidural anesthesia (EDA) result in a redistribution of warm blood from the body core to the periphery [3]. Furthermore, the induction of general anesthesia (GA) aggravates redistribution hypothermia up to 1.5 °C [4]. Cytoreductive surgery for ovarian cancer is characterized by maximum extent of tumour debulking including deperitonea-lisation and massive volume shifts and losses [5]. Stabilisation of the BCT, therefore, requires forced air warming and administration of warmed infusions and transfusions. However, maintainance of normothermia is jeopardized by an anesthesia-reduced base metabolic rate and heat production [5]. Perioperative hypothermia may affect coagulation by altered platelet function [6, 7] and increase intraoperative blood loss [8]. Furthermore, hypothermia-related vasoconstriction may impair microperfusion and reduce the oxygen partial pressure and oxygen saturation (StO_2_) in peripheral tissues [9]. As a result of impaired microperfusion cell metabolism of subcutaneous fat- or skeletal muscle cells, measured in microdialysis (MD), may be affected [10].

Preoperative forced-air warming (prewarming) of 10 to 60 min at 43 °C [11–15] before induction of GA has been shown to reduce the temperature gradient between body core and periphery [16] in order to alleviate redistribution hypothermia [3, 16] and to maintain intraoperative normothermia [17] in minor surgery (without EDA) [11] or major abdominal surgery with EDA [12]. However, these studies required holding areas to realize prewarming and a timely patient flow. From a surgical point of view, in major cytoreductive surgery with a duration of more than 180 min it still remains unclear whether a brief prewarming during insertion of EDA is effective to maintain intra- and postoperative normothermia and to improve intraoperative hemodynamics and microperfusion as well as to reduce postoperative shivering and ventilation [18].

We hypothesized that a forced-air prewarming interval of 30 min at 43 °C during epidural catheter placement and induction of GA without any changes of the perioperative patient flow (e.g. no treatment in a preoperative holding area) is effective to maintain perioperative normothermia and improve microperfusion during cytoreductive surgery for ovarian cancer.

## Methods

This single-center prospective randomized clinical trial was conducted at the Departments of Anesthesiology and Intensive Care Medicine and Gynecology of the Charité-Universitätsmedizin Berlin, Germany. Ethics approval was obtained from the institutional ethics committee (EA1/348/14, ClinicalTrials.govID: NCT02364219). Women were enrolled if they were of age (> 18 years), mentally healthy, American Society of Anesthesiologists physical status Class I–III and scheduled to undergo elective major cytoreductive surgery in primary or secondary ovarian cancer under combined epidural and general anesthesia. Exclusion criteria were: heart failure (left ventricular ejection fraction (LV-EF) < 30%), chronic obstructive pulmonary disease (GINA-Classification > 3), renal failure (glomerular filtration rate (GFR) < 50 ml/min) or dialysis, participation in another clinical trial and contraindication of EDA including non-eligibility due to refusal of neuraxial anesthesia.

After written informed consent and before surgery, women were prospectively randomized into prewarming (prewarm) and standard group (standard) using a computer-generated block randomization (Excel, Microsoft Corporation, Redmont, WA, USA). Additionally, a subgroup of the standard- and the prewarm group were randomly assigned for microdialysis (MD) measurements. The randomization was concealed. Randomization was blinded, the anesthesist during surgery was not randomized and the data evaluation took place independently from the surgery anesthesist.

**Fig. 1:**
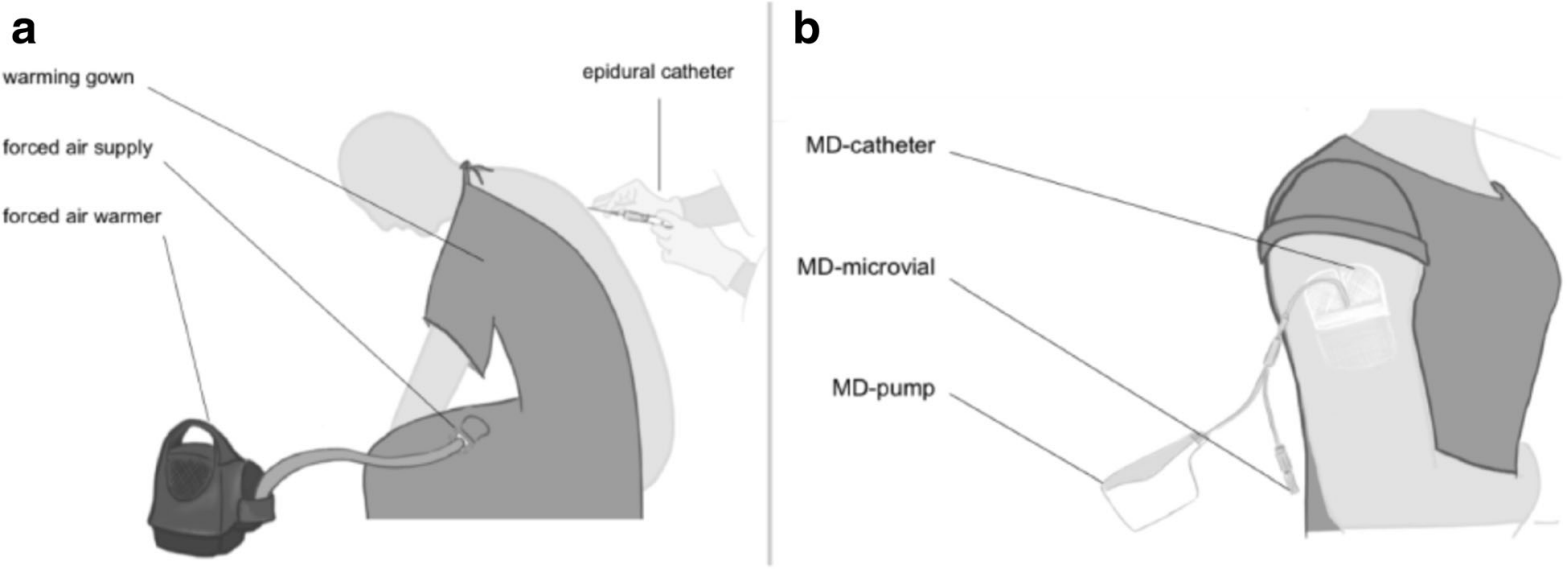
**a**) Forced air prewarming during insertion of the epidural catheter; **b**) subcutaneous microdialyses in the upper arm

According to randomization, women in the prewarming group received forced-air warming at 43 °C, during the establishment of epidural anesthesia. Prewarming was applied using a forced air warming gown (Bair Paws™ Flex Warming Gown, 3 M™, St. Paul, MN, USA) connected to a forced air warmer (Bair Hugger™, Model 750, 3 M™, St. Paul, MN, USA), which warms the front of the body including upper arms (Fig. 1a). In the standard treatment group, patients were covered by a cotton blanket for passive insulation without active warming during the insertion of the epidural catheter.

The perioperative anesthetic setting in both groups was based on the clinical practice and standard operating procedures of the Department. Upon arrival in the operating room (OR), a peripheral vein cannula was inserted and an infusion of balanced crystalloid solution was started under standard monitoring. Body core temperature was taken by using the SpotOn™ temperature system (3 M™; St. Paul, MN, USA) in both groups and throughout the observation period [19]. Perioperative body core temperatures were manually recorded in the case reporting form (CRF) at admission to the OR (T_1_), insertion of epidural catheter and induction of GA (T_2_) as well as after 15 min (T_3_), 60 min (T_4_), 120 min (T_5_), 180 min (T_6_), at the end of operation (T_7_), on admission to the ICU (T_ICU_) and 2 h postoperatively (T _ICU2h_). In both groups, a thoracic epidural catheter was inserted in the sitting position (Fig. 1a). After induction of GA, EDA was established by a bolus followed by a continuous rate of ropivacaine/ sufentanil (CADD™ Solis pump, Smiths Medical, St. Paul, MN, USA). GA was induced using propofol, fentanyl and rocuronium for endotracheal intubation and maintained with sevoflurane with a minimum inspired oxygen concentration of 40% and an end-tidal CO_2_ concentration of 35 to 40 mmHg. Anaesthesia and muscle relaxation was maintained with repetitive bolusses of fentanyl and rocuronium. A central venous line (Arrow Medical Limited, Kington, UK) was placed into the internal jugular vein and blood pressure was measured by an arterial line (Vygon, Ecouen, France). During surgery, all intravenous (i.v.) fluids were infused using an active warming device (Level 1, Hot Line™, Smiths Medicals, St. Paul, MN, USA). Women were warmed by forced air warming of the upper part of the body (Standard-group: 3M™ Bair Hugger™ Intraoperative Blanket; PW-group: 3M™Bair Paws™ Flex Warming Gown) at 43 °C until end of anesthesia. Forced air warming was shortly paused in both groups during disinfection of the belly before surgery. Postoperatively, women were transferred to the post-anesthesia care unit (PACU). If patients showed clinical signs of hypovolemia (such as heart rate (HR) > 100 bpm, mean arterial pressure (MAP) < 60 mmHg, increased pulse pressure variation (PPV)) as well as hypovolemic changes in the hemodynamic monitoring (stroke volume variation (SVV), stroke volume (SV) and CO; Flo-Trac™, Edwards Lifesciencess, Irvine, CA, USA) and in case of intraoperative volume- and blood loss, crystalloid infusion, gelatine infusion (500–1000 ml Gelafundin™ 4% BBraun, Melsungen, Germany) and, if needed, fresh frozen plasma (FFP) and red blood cell concentrates (RBC) were given according to the transfusion- and hemodynamic algorithm of the standard operating procedures (SOP) of the Department of Anesthesiology and Intensive Care Medicine [20]. During surgery, StO_2_ was measured by near-infrared spectroscopy (InSpectra Spot Check, M300™, Hutchinson, MN, USA) on the thenar of the right hand. Central venous and arterial blood gas analysis to determine dCO_2_, lactate and hemoglobin (Hb) were performed every 30 min. Patients were extubated when spontaneous breathing was sufficient, body core temperature > 35.5 C and no clinical symptoms of hypovolemia or hemodynamic instability. Postoperative pain relief was achieved using a patient-controlled epidural analgesia (PCEA) and women were transferred to PACU for at least 24 h after operation [20].

For MD, a 60 mm (inlet tubing diameter 1 mm) flexible catheter (63 MD catheter, molecular weight cut-off of 2kD, M Dialysis AB, Stockholm, Sweden) was placed into the subcutaneous fat tissue below the right deltoid muscle after local anesthesia (Fig. 1b). After perfusion with isotonic sterile fluid (perfusion fluid T1, M Dialysis AB) at a rate of 1 μl/min (MD Pump 106, M Dialysis AB) a dialysate was collected every 30 min (first sample at T_3_) in a 200 μl microvial (M Dialysis AB) and stored at − 20 °C until analysis. At the end of surgery, the catheter was removed while the patient was still unconscious. All MD analysis were performed by the Experimental & Clinical Research Center (ECRC) of the Franz-Volhard-Centrum, Charité-Universitätsmedizin Berlin, Campus Berlin-Buch.

The primary endpoint was defined as body core temperature drop (BCT_drop_) from before epidural catheter placement (T_1_) and after induction of GA (T_2_) in the prewarming or standard group. Intra- and postoperative change of BCT up to 2 h after ICU admission, StO_2_ and hemodynamic parameters (PPV, SVV, SV), MAP, HR, norepinephrine usage, intraoperative transfusion rates (FFP, RBC), arterial lactate as well as the ethanol ratio, the glucose-, lactate- and glycerol concentration via MD were defined as secondary endpoints. In addition, the central venous-arterial dCO_2_, which depends on the CO_2_ production of the tissues and CO, were measured as a marker for microperfusion [21]. Postoperatively, shivering and postoperative ventilation were obtained.

Sample size calculation was based on the assumption of a minimal difference of 0.5 °C in body core temperature after prewarming compared to no prewarming (standard) [11]. A sample size of totally 48 patients, divided into two groups, was calculated to provide 80% power for detecting statistically significant difference at a two-tailed significance level of 0.05 (nQuery Advisor**™**, Release 7.0, Stat. Solutions Ltd. & South Bank, Crosse’s Green, Cork, Ireland). A dropout rate of 10% of patients not eligible for data sampling was included.

Data are reported as arithmetic mean [standard deviation (SD)] or median [25, 75% percentile] (after rejection of normal distribution) or frequencies (%). In order to assess differences in body core temperature as the primary outcome at timepoints T_1_-T_7_ the nonparametric (exact) Mann-Whitney-U test was used. Differences between the groups in terms of patient characteristics as well as secondary outcomes were compared with paired or unpaired t-test and chi-squared test. Frequencies were tested by the exact Mantel-Haenszel test (for ordered categories) or the exact Chi-square test (for unordered categories). A two-tailed *p*-value of < 0.05 was considered statistically significant.

For MD subgroup analysis a sample size of 7 patients for each group was defined according to the study of Rosdahl et al. [10].

All calculations were performed with R software (version 3.3; R Foundation for Statistical Computing, Vienna, Austria).

The reporting of this RCT follows the updated guidelines for reporting parallel group randomized trials (CONSORT 2010 statement).

## Results

**Fig. 2:**
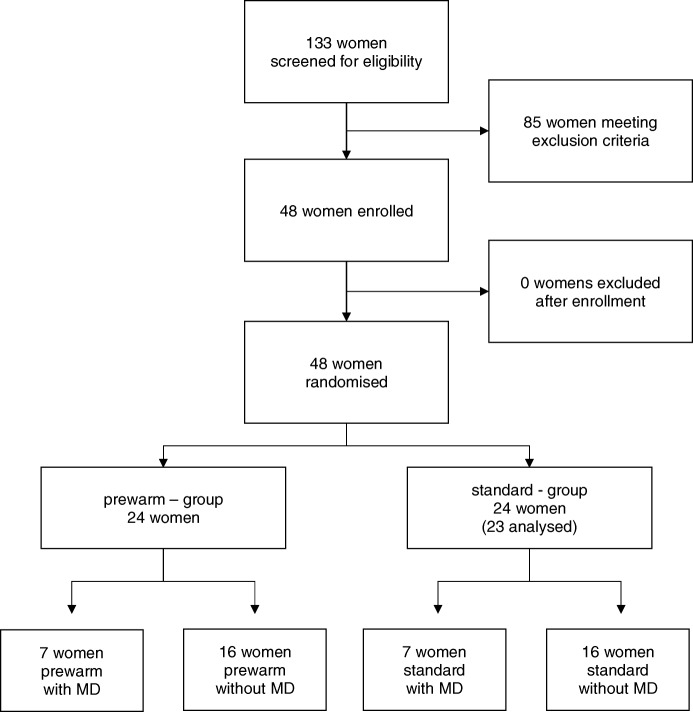
CONSORT flow diagram

During January 2015 and December 2017 133 women were screened for eligibility. 85 patients did not meet the inclusion criteria (Fig. 2) and were excluded before randomization, leaving 48 women for randomization (Fig. 2). Data of 47 women were analyzed, as one woman had to be excluded due to termination of the procedure out of surgical reasons (Fig. 2).

Besides a slight difference in height (*p* = 0.047), patient baseline characteristics, comorbidities and the duration of surgery did not differ significantly between the groups (Table 1). In multivariable linear regressions with group and BMI, group and age or group, BMI, and age as independent variables only group showed a significant relationship (*p* < 0.05) with body core temperature change.

**Table 1:** Patient baseline characteristics, comorbidities and duration of surgery

	prewarm *n*=24		standard *n*= 23	
Age (Years, mean±SD)	54,4	(±11,7)	61,3	(12,1)
Weight (kg, mean±SD)	73,6	(17,6)	67,8	(10,3)
Height (cm, mean±SD)	167	(4,9)	164,0	(6,1)*
BMI (mean±SD)	26,3	(5,8)	25,4	(4,1)
FIGO stage (n, %)				
I	1	(4)	0	(0)
II	0	(0)	0	(0)
III	17	(68)	14	(64)
IV	5	(20)	4	(18)
Residual tumor after surgery (n, %):				
0 cm	18	(72)	17	(77)
0-1 cm	5	(20)	4	(18)
1-2 cm	1	(4)	1	(5)
>2 cm	2	(8)	0	(0)
Preoperative fasting (Liquids in hrs, mean±SD)	11,3	(4,2)	11,6	(3,8)
Preoperative fasting (Solid in hrs, mean±SD)	16,7	(4,7)	16,2	(4,8)
Coronary artery disease (n,%)	0	(0)	1	(4,4)
Heart failure (n,%)	0	(0)	0	(0)
Diabetes mellitus (n,%)	1	(4,2)	1	(4,4)
Hypothyroidism (n,%)	1	(4,2)	4	(17.4)
Renal failure (n,%)	0	(0)	1	(4,4)
Arterial hypertension (n,%)	5	(20,8)	7	(30,4)
Peripheral arterial vascular disease (n,%)	1	(4,2)	1	(4,4)
COPD (n,%)	1	(4,2)	1	(4,4)
Asthma (n,%)	3	(12,5)	2	(8,7)
Thrombosis (n,%)	3	(12,5)	1	(4,4)
Hyperthyroidism (n,%)	1	(4,2)	1	(4,4)
Nicotine abuse (n,%)	0	(0)	1	(4,4)
Hyperlipoproteinemia (n,%)	0	(0)	3	(13)
ASA Classification (n,%):				
1	4	(16,7)	3	(13)
2	12	(50)	14	(60,9)
3	8	(33,3)	6	(26,1)
Duration of surgery^§^ (min, mean ± SD)	334	±114	364	±134

*p < 0.05; ^§^ incision to suture; *SD* Standard deviation, *BMI* body mass index, *FIGO* International Federation of Gynecology and Obstetrics, *COPD* chronic obstructive pulmonary disease, *ASA* American Society of Anesthesiologists

**Fig. 3:**
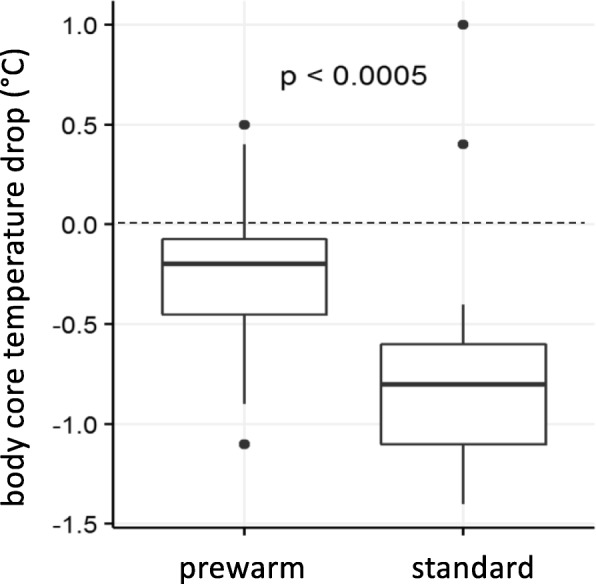
Primary endpoint: Body core temperature drop between T_1_ and after induction of general anesthesia T_2_ with (prewarm) or without (standard) prewarming [median and quartile (25%; 75%)]

**Fig. 4:**
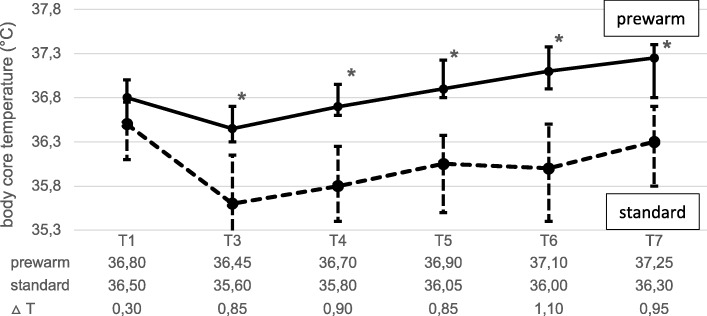
Secondary endpoints: Body core temperature before prewarming (T_1_) and during operation (T_3_ to T_6_) and at the end of surgery (T_7_) [median and quartile (25%; 75%); * *p* < 0,001]

**Fig. 5:**
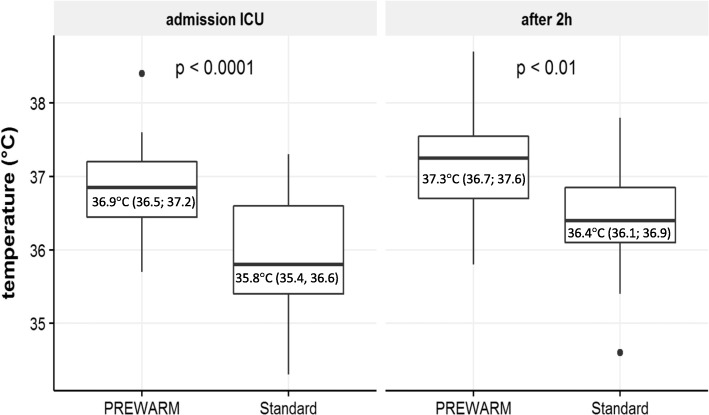
Secondary endpoints: Body core temperature at T_ICU_ and T_ICU2h_

Regarding the primary endpoint, the body core temperature change from T_1_ (before) to T_2_ amounted to 0.35 °C in the prewarm group and 0.9 °C in the standard group (Fig. 3) with a significant difference (ΔT) at T_2_ = 0.55 °C (*p* < 0.005) between both groups (Fig. 3). No significant difference in baseline body core temperature (T_1_) was measured (Fig. 4). After a further decrease of the BCT between T_2_ and T_3_ with a significant ΔT_3_ of 0.85 °C (*p* < 0,001) (Fig. 4) the BCT increased in both groups during the operation (Fig. 4). The core temperature remained higher in the prewarm group over the entire observation period with a significant ΔT at all time points from (ΔT_4_ = 0.9 °C up to ΔT_7_ = 0.95 °C, *p* < 0,001, Fig. 4). At T_ICU_ and at T_ICU2h_ the body core temperatures in the PREWARM group were also higher than in the standard group (Fig. 5). One woman in the prewarm group suffered from hypothermia (< 36 °C) temporarily while 17 women in the standard group were hypothermic at one time point and/or over the entire intraoperative observation period (*p* < 0,001). At T_ICU_ six women in the standard group, but none in the prewarm group were subject to postoperative ventilation (*p* < 0.001) due to hypothermia at a median core temperature of 34.9 °C (34.8; 35.0). Three women in the standard group at T_ICU_ only, but none in the PREWARM group experienced postoperative shivering (*p* > 0.05).

StO_2_ increased from 86% (T_3_) to 88% (T_7_) in the prewarm group and was slightly, but not significantly, higher compared to 82% (T_3_) and 85% (T_7_) in the standard group. In both groups, the dCO_2_ did not change intraoperatively (median: prewarm 6.25 mmHg; standard 6.15 mmHg). Hemodynamic parameters such as SVV, SV and PPV as well as MAP, HR and norepinephrine concentration varied temporarily, depending on the volume changes in both groups, but without any significant differences between both groups at T_3_, T_4_ and T_5_ (Table 2). FFP- and RBC transfusion were used in both groups with a non-significant difference between the groups (Table 2). The intraoperative arterial lactate concentration varied within the scope of 6 mg/dl (T_3_) to 7.5 mg/dl (T_7_) without significant differences between both groups.

**Table 2:** Secondary endpoints: Intraoperative hemodynamic parameters at T_3–5_ and total amount of transfusion at the end of surgery

	prewarm [mean; (SD)]		standard [mean; (SD)]	
MAP (mmHg)				
T_3_	80	(±12)	77	(±18)
T_4_	77	(±10)	74	(±17)
T_5_	78	(±9)	75	(±17)
HR (bpm)				
T_3_	62	(±13)	60	(±15)
T_4_	68	(±12)	65	(±15)
T_5_	72	(±16)	71	(±19)
SVV (%)				
T_3_	9	(±3)	8	(±3)
T_4_	9	(±3)	9	(±3)
T_5_	9	(±3)	9	(±3)
SV (ml)				
T_3_	69	(±12)	67	(±16)
T_4_	74	(±18)	73	(±21)
T_5_	81	(±21)	77	(±24)
PPV (%)				
T_3_	7	(±3)	7	(±3)
T_4_	9	(±3)	8	(±3)
T_5_	9	(±5)	9	(±5)
Norepinephrine (μg/kg/min)				
T_3_	0.03	(± 0.02)	0.03	(±0.02)
T_4_	0.06	(±0.04)	0.06	(±0.04)
T_5_	0.07	(±0.05)	0.07	(±0.04)
Transfusion (ml)				
FFP at T_7_	3500	(±2300)	3700	(±2200)
RBC at T_7_	1000	(±600)	1000	(±900)

*p < 0.05; *MAP* mean arterial pressure, *HR* heart rate, *SVV* Stroke volume variation, *SV* Stroke volume, *PPV* pulse pressure variation, *FFP* fresh frozen plasma, *RBC* red blood cells concentrates

**Fig. 6:**
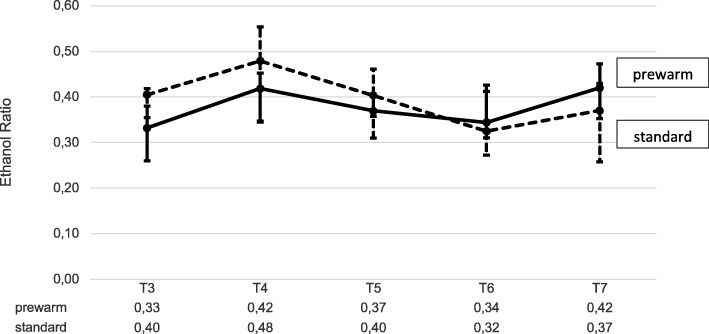
Secondary endpoints: Ethanol ratio in MD before prewarming (T_1_) and during operation (T_4_to T_6_) and at the end of surgery (T_7_) [median and quartile (25%; 75%)]

For the ethanol ratio, the glucose-, lactate- and glycerol concentration [median (25. quartile/75. quartile)] measured by MD from T_3_ to T_7_, no significant differences were detectable between the groups. On closer view, the ethanol ratio (Fig. 6) was temporarily reduced in the prewarm compared to the standard group, with comparable results at the end of surgery (T_6_). The glucose concentration deferred from 2.05 (0.36/0.36) mmol/l in the prewarm group versus 1.02 (0.42/0.59) mmol/l in the standard group at T_3_ and proceeded almost similar in both groups between 1.51 (0.44/0.87) and 2.1 (1.35/0.35) mmol/l from T_4_ to T_7_. The glycerol concentration decreased continuously from 315 (163/278) μmol/l at T_3_ to 191 (21/185) μmol/l at T_7_ in the prewarm group and from 245 (106/187) μmol/l at T_3_ to 119 (52/131) μmol/l at T_7_ in the standard group. The lactate concentration of 0.66 (0.17/0.07) mmol/l at T3 in the prewarm group was higher compared to 0.48 (0.25/0.18) mmol/l in the standard group and both concentrations aligned at 0.6 (0.17/0.02) mmol/l at T_6_.

## Discussion

In this study a forced-air prewarming interval at 43 °C during epidural catheter placement and induction of GA showed efficacy to reduce core temperature drop and to maintain intraoperative normothermia during cytoreductive surgery not only throughout extended surgery but also at ICU admission and two hours thereafter. Furthermore, prewarming reduced the risk of postoperative ventilation due to hypothermia at the end of surgery. Microdialysis and StO_2_ showed better results for the prewarming group, however, this did not reach statistical significance. A reason for this may have been, that all hemodynamic parameters, transfusion rates, and dCO_2_ were not different in either study group. Nevertheless, small sample size for secondary outcomes should be taken into account for further discussions.

In a prospective randomized trial in 99 patients undergoing major abdominal surgery, Horn et al. [12] analyzed the effects of prewarming over 15 min before and after EDA versus 15 min after EDA or no prewarming. In contrast to our study, forced-air prewarming was performed at 44 °C in a preoperative care unit before and after EDA, which requires higher logistical and staff demands than our approach [12]. Nevertheless, despite pre- and intraoperative forced-air warming the drop in body core temperature with induction of GA still occurs, which can be significantly alleviated by prewarming, which is in line with earlier results [12, 22].

At ICU admission only six women of the standard group but none in the prewarming group were mechanically ventilated on ICU because of hypothermia. These findings are in line with the findings of Horn et al. who measured a 34% postoperative ventilation rate in non-prewarmed patients, which indicates the importance of temperature management for ventilation times after surgery. Even 2 h after ICU admission prewarmed women of our study had a higher body core temperature without any shivering. Our data suggest, that the greater body core temperature drop after induction of GA in the standard seems to be irreversible in spite of all intraoperative approaches, such as intraoperative forced air warming, warm fluids etc., to warm up the patients during surgery effectively.

We hypothesized that StO_2_, dCO_2_, arterial lactate and parameters of MD may be useful as surrogate parameters to measure an improved microperfusion in prewarmed and normothermic patients. These markers did not differ significantly, but showed a trend in favor of the prewarm group, which may indicate the need for further studies powered to detect a difference in microcirculation parameters.

We measured a non-significant difference in StO_2_ in favor of the prewarmed patient group_._ It still remains unclear if this effect is caused by mild hypothermia-induced vasoconstriction and consecutive hypoperfusion and could be prevented by measures to maintain normothermia [23].

Our MD data could suggest that the tendency of a temporarily reduced ethanol ratio in prewarmed women after induction of GA and during the first hours of operations may represent an improved tissue perfusion [10]. After normothermia is reestablished due to intraoperative forced-air- warming and warmed transfusion/iv-fluids, the ethanol ratios showed similar values. This is consistent with the other microdialysis parameters. In 13 healthy volunteers, Rosdahl et al. demonstrated that the availability of glucose as a substrate for cell metabolism in subcutaneous fat tissue is dependent on tissue perfusion [10]. Therefore, the glucose recovery rate in MD acts approximately inverse to the ethanol ratio [10]. This might explain the increased glucose concentration in the prewarmed group at the beginning of surgery when perfusion may be improved due to the higher body core temperature compared to the standard group. Moreover, an increase of the lactate concentration in MD represents hypoperfusion and oxygen deficit in the subcutaneous tissue, as Bahlmann et al. demonstrated after 15–60 min of aortic clamping [24]. Potential hypoperfusion of the subcutaneous tissue due to reduced body core temperature did not result in an increase of the lactate concentration in the standard group with MD. On the other hand, the glycerol concentration in MD represents increased metabolic stress and lipolytic activity in the standard group [24]. Unfortunately and similar to lactate, the concentration remained without significant changes and differences between the groups. It may be hypothesized that mild hypothermia or reduced body core temperature are not very likely to result in a detectable increase of lactate and glycerol in MD. Nevertheless, the MD parameters give a first impression of temperature-related effects on microperfusion and that MD may be a promising technique to identify surrogate parameters for the impact of hypothermia. So far, no comparable data of MD in dependence of changes in body core temperature are published.

## Limitations

Our study has a few limitations: Firstly, the study was powered in regards to primary outcome. In terms of secondary outcome, the study was not powered to detect significant changes, but may allow for an estimation of the number of patients needed for upcoming investigations on microperfusion and MD. Secondly, StO_2_ was evaluated without reference values of an accompanied vascular occlusion test on the upper arm due to potential disruption and damage to the microdialysis catheter, which was inserted on the same side. Lastly, prewarming did not allow a blinding at all times. A concealed randomization was performed. Furthermore the anesthesiologist in charge of the patient was not involved in the collection of study related data. So the likelihood of bias was reduced.

## Conclusion

In conclusion, forced-air prewarming at 43 °C during epidural catheter placement and induction of GA without interfering with the perioperative routine setting is effective to reduce the core temperature drop. Furthermore, prewarming maintains intraoperative normothermia until the end of surgery and up to two hours after ICU admission without any shivering or mechanically ventilation due to hypothermia postoperatively. A statistically significant impact of prewarming on microperfusion as measured by MD could not be detected by this study. However, reduced ethanol ratio and increased glucose concentration in the microdialysis as well as an increased tissue oxygenation may point to a better maintained microperfusion in prewarmed patients compared to those receiving standard treatment. The use of hemodynamic parameters, transfusion rates, StO_2_, dCO_2_ and MD parameters as surrogate parameters influencing microperfusion should be controlled in further randomized, controlled studies investigating the impact of temperature management in surgical patients.

## Data Availability

The datasets used and analyzed during the current study are available from the corresponding author on reasonable request. The complete trial protocol for this investigator-initiated trial is available in German only and can be requested by the corresponding author.

## Abbreviations

BCT: body core temperature
BCT_drop_: drop in body core temperature
CO: cardiac output
CRF: case reporting form
dCO_2_: carbon dioxide difference
ECRC: Experimental & Clinical Research Center
EDA: Epidural anesthesia
FFP: fresh frozen plasma
GA: General anesthesia
GFR: glomerular filtration rate
GINA: Global Initiative for Asthma
HR: heart rate
i.v.: intravenous
ICU: Intensive Care Unit
LV-EF: left ventricular ejection fraction
MAP: mean arterial pressure
MD: microdialysis
OR: operating room
PACU: post-anesthesia care unit
PCEA: patient-controlled epidural analgesia
PPV: pulse pressure variation
PW: prewarming
RBC: red blood cell concentrates
SD: standard deviation
SOP: standard operating procedures
StO_2_: tissue oxygenation
SV: stroke volumen
SVV: stroke volume variation
T_1_: Temperature at time before induction of anesthesia
T_2_: Temperature after induction of anesthesia
T_3_: Temperature after 15 min of induction of anesthesia
T_4_: Temperature after 60 min of induction of anesthesia
T_5_: Temperature after 120 min of induction of anesthesia
T_6_: Temperature after 180 min of induction of anesthesia
T_7_: Temperature at the end of operation
T_ICU_: Temperature on admission to the ICU
T_ICU2h_: Temperature until 2 h after ICU admission
